# iTRAQ-Based Proteomics Reveals Novel Biomarkers for Idiopathic Pulmonary Fibrosis

**DOI:** 10.1371/journal.pone.0170741

**Published:** 2017-01-25

**Authors:** Rui Niu, Ying Liu, Ying Zhang, Yuan Zhang, Hui Wang, Yongbin Wang, Wei Wang, Xiaohui Li

**Affiliations:** 1 Department of Respiratory Medicine, Second Hospital of Shandong University, Shandong, China; 2 Operating Room, Tianjin Chest Hospital, Tianjin, China; 3 Department of Evidence-based Medicine, Second Hospital of Shandong University, Shandong, China; 4 Department of Nursing, Second Hospital of Shandong University, Shandong, China; Helmholtz Zentrum München, GERMANY

## Abstract

Idiopathic pulmonary fibrosis (IPF) is a gradual lung disease with a survival of less than 5 years post-diagnosis for most patients. Poor molecular description of IPF has led to unsatisfactory interpretation of the pathogenesis of this disease, resulting in the lack of successful treatments. The objective of this study was to discover novel noninvasive biomarkers for the diagnosis of IPF. We employed a coupled isobaric tag for relative and absolute quantitation (iTRAQ)-liquid chromatography–tandem mass spectrometry (LC–MS/MS) approach to examine protein expression in patients with IPF. A total of 97 differentially expressed proteins (38 upregulated proteins and 59 downregulated proteins) were identified in the serum of IPF patients. Using String software, a regulatory network containing 87 nodes and 244 edges was built, and the functional enrichment showed that differentially expressed proteins were predominantly involved in protein activation cascade, regulation of response to wounding and extracellular components. A set of three most significantly upregulated proteins (HBB, CRP and SERPINA1) and four most significantly downregulated proteins (APOA2, AHSG, KNG1 and AMBP) were selected for validation in an independent cohort of IPF and other lung diseases using ELISA test. The results confirmed the iTRAQ profiling results and AHSG, AMBP, CRP and KNG1 were found as specific IPF biomarkers. ROC analysis indicated the diagnosis potential of the validated biomarkers. The findings of this study will contribute in understanding the pathogenesis of IPF and facilitate the development of therapeutic targets.

## Introduction

Idiopathic pulmonary fibrosis (IPF), a devastating form of chronic interstitial lung diseases with an unfavorable outcome leading to respiratory failure, is a chronic fibrosing interstitial pneumonia encountered by clinicians in the intensive care units [1, 2]. Since its description, significant research has been achieved to advance our knowledge about its pathogenesis, epidemiology and treatment options. Nevertheless, there are no hitherto established drug therapies because suitable symptoms for initiating therapy, best candidates for treatment, and conceivable role for combinatory therapies are still controversial [3, 4]. The mortality from IPF is nearly 40% with a median survival of 2 to 3 year [5, 6]. This high mortality is partly due to incomplete understanding of the molecular mechanisms that are specifically lacking in the IPF patients who do not recover from their illness. To address this gap in knowledge, it is essential to characterize the protein expression in order to identify the proteins and the biological processes that are different in the IPF patients vs. healthy subjects in order to facilitate earlier and correct diagnosis of IPF for establishing effective treatment and prolonging survival.

Advances in molecular techniques have largely contributed in our interpretation of IPF and have permitted the identification of new metabolic pathways and few IPF treatment targets with hypothetically novel anti-fibrotic agents [7–10]. Up to date, limited studies [7–16] have proposed invasive and non-invasive biomarkers for IPF using genomics and transcriptomics approaches. Protein biomarkers are being developed as clinically valuable biomarkers for diverse diseases, and the easy access to human blood has prompted serum proteins as non-invasive biomarkers in clinical sets. Thus, the documentation of differentially expressed serum proteins would facilitate the development of biomarkers for IPF diagnosis and prognosis.

Proteomics tools allow the high throughput studies of protein expression, and thereby enables the identification of molecular mechanisms that are responsible for the development of specific diseases [17]. Compared to RNA-seq or microarray-based profiling of gene expression, which have been previously applied to IPF, proteomics analysis offers various advantages [18, 19]. In particular, proteomics indicates the effective existence of functional proteins in studied samples. In addition, high transcriptional level producing abundant quantity of mRNA does not imply the high amount of the corresponding protein or its effectual activity [20]. Therefore, using proteomics is significantly strategic in unearthing specific diagnostic biomarkers and new therapeutic targets.

A coupled iTRAQ/LC–MS/MS-based approach has been applied as a powerful sensitive proteomics tool for concurrently quantifying proteins in 4- or 8-plex samples during the discovery of disease biomarkers [21]. However, to the best of our knowledge, there is no consequential study on the use of iTRAQ-based approaches in the identification of serum biomarkers for IPF.

In this study, we aimed to characterize protein expression in serum from patients with IPF to identify the differences in the protein expression between IPF patients and healthy individuals in order to provide insights into the pathways and biological processes that differ in the two comparison groups and identify potential biomarkers for discriminating between IPF patients and healthy individuals.

## Methods

### Ethics statement

The Ethical Review Board of Research Projects involving Human Subjects of the Second Hospital of Shandong University approved this study. Written informed consent for participation in the study was obtained from either the patient or the patient’s legal representative who had to fill in a patient questionnaire in line with the declaration of Helsinki prior to patients’ enrollment.

### Initial study population

IPF Patients (n = 60, 60–87 years old) and healthy subjects (n = 60, 40–67 years old) were recruited at the Second Hospital of Shandong University from January 2014 to December 2015. For this study, subjects were randomly distributed in six groups (three groups of healthy individuals and three groups of IPF patients with 20 individuals in each group). The diagnosis of IPF was based on published consensus guidelines [22] and the “Guidelines for Diagnosis and Management of Idiopathic Pulmonary Fibrosis” established by the Chinese Medicine Association (2002) and determined on the basis of high-resolution computed tomography (HRCT) or surgical lung biopsy showing a definite usual interstitial pneumonia pattern. The most common symptoms at the onset of the disease were irritating cough, shortness of breath, dyspnea, loss of endurance activities and other symptoms of dyspnea. The anomalies of pulmonary function included restriction of ventilation function and impairment of lung diffusion. The X film of the chest showed a reticular shadow in the surrounding lung area, which occurred mainly in the basal part of the lung. The main HRCT results showed double lung bottom patch or mesh like changes, with varying degrees of grinding glass-like shadow. The healthy controls were selected after a rigorous physical examination at the Physical Examination Center of the Second Hospital of Shandong University. Baseline clinical and demographic statistics of IPF and control subjects is indicated in Table 1.

**Table 1:** Clinical and demographic IPF variables categorized by forced vital capacity (FVC, (%)) and diffusing capacity for carbon monoxide (D_L_CO (%)).

Variable	Characteristics	IPF (N = 60)	Healthy control (N = 60)
**Predicted FVC (%)**		79±10%	100%
**Predicted DLCO (%)**		68±8%	100%
**Age (years)**	Mean±SD	72.5±10.56	67.98±8.48
	Range	60–87	49–80
**Sex**	Male	40	36
	Female	20	24
**Smoking status**	Current smokers	0	0
	Former smokers	11	0
	Never smoked	40	60
	Not reported	9	0

### Independent validation study population

For validation of the iTRAQ/LC-MS-MS data, we collected serum samples from 20 IPF patients, 20 hypersensitivity pneumonitis patients, 20 sarcoidosis patients and 20 healthy control individuals at the Second Hospital of Shandong University from January 2014 to December 2015 for ELISA test. The diagnosis of IPF was as described above while patients with sarcoidosis were confirmed according to the diagnostic criteria established by the European Respiratory Society criteria and American Thoracic Society [23]. The average FVC% and DL_CO_% of sarcoidosis patients were 71.8 ± 15.3 and 69.75 ± 20.4, respectively. The clinical diagnosis of patients with hypersensitivity pneumonitis (HP) according diagnostic criteria established in previous studies [24, 25]. The average FVC% for HP was 65.98 ± 5.7.

### Blood collection

From each fasting subjects, 5 mL of peripheral blood were collected into a BD Vacutainer tube without anticoagulant using standardized phlebotomy procedures. After centrifugation at 10,000 rpm for 10 minutes at 4°C, all samples were rapidly aliquoted and stored at -80°C until analysis. Only samples obtained before treatment of IPF patients were employed for proteomic analysis. All serum samples from IPF patients and healthy individual groups were randomized, and the researcher was blinded to their characteristics.

### Protein extraction and quantification

Proteins were extracted from serum samples at GUANGZHOU FITGENE BIOTECHNOLOGY co.Ltd in December 2015 according to the protocol described in the ProteoPrep Blue Albumin and IgG Depletion Kit (Sigma). Briefly, 400 μL resin homogenate was filtered into a 2 mL tube and then centrifuged at 10000 rpm for 10 minutes. After removal of the preservation solution, 400 μL of equilibration solution was added for two washings of the resin homogenate. After that, 80 μL of pooled serum samples (3 pools from the 3 control groups and 3 pools from the 3 IPF patient groups) were slowly added onto the resin and centrifuged at 10000 rpm for 10 min. Subsequently, samples were collected and added to the resin for 10 min adsorption, followed by centrifugation at 10000 rpm for 10 min. Thereafter, 100 μL of equilibration solution was added and followed by subsequent centrifugation at 10000 rpm for 10 min. The supernatant filtrates were mixed with 4 volumes of acetone, and stored at -20°C overnight for protein precipitation. The precipitated proteins were centrifuged at 4°C and 12000 rpm for 20 min. Finally, the supernatant was removed and the protein extracts air-dried and stored at -80°C.

Protein concentration was measured using the QuantiPro™ BCA Assay Kit (Sigma, catalogue: QPBCA). Each tube was added with 100 μl of deionized water, 100 μl of BCA solution and vortexed for 20 s. The reaction was done at 60°C and lasted for 1 h after which the absorbance was measured at 575 nm using a spectrophotometer purchased from SHANGHAI Spectrum Co, Ltd. The quantification was performed using a standard calibration curve generated for this purpose. Protein extraction and quantification were repeated as necessary to collect a total of 200 μg protein for each group.

### Protein digestion and iTRAQ labeling

Prior to the iTRAQ labeling experiments, equal quantities of 200 μg of proteins extracted as described above were trypsinised according to the FASP protocol [26]. After an overnight digestion, peptides were eluted from the filters with 25 mM ammonium bicarbonate buffer. Eluted peptides were concentrated and purified on C18 StageTips.

Subsequently, iTRAQ labeling was performed using an 8-plex iTRAQ labeling kit (AB Sciex, catalogue: 4381664) according to the manufacturer's protocol. The iTRAQ labeling was performed as follows: 3 controls (non-IPF pools) were respectively labelled with iTRAQ reagents 113, 115 and 117 whereas 3 IPF samples were labelled with iTRAQ reagents 114, 116 and 119. The concentrated iTRAQ Reagent-Labeled Digest Samples from the six groups were next solubilized in 250 μL of loading buffer A (20 mM ammonium formate, pH 10) and combined into one tube prior to fractionation.

### High pH One-D reverse phase chromatography fractionation

Fractionation of the peptide mixture was performed on a Dionex Ultimate 3000 HPLC system equipped with an UltiMate 3000 RS pump, an UltiMate 3000 RS column compartment and an UltiMate 3000 RS autosampler (Dionex, Olten/Switzerland). The column was initially equilibrated for 70 min in buffer A (20 mM ammonium formate, pH 10) as described above before sample injection. The tryptic digest was injected onto a Phenomenex columns (Gemini-NX 3u C18 110A; 150*2.00mm) using a linear gradient increasing at a rate of 1% B per minute from 2–45% mobile phase B (A: 20 mM HCOONH4 at pH 10; B: 20mM HCOONH4, 80% CAN at pH 10) at a flow rate of 200μl/min while monitoring a UV absorbance at 214 nm/280 nm. Fractions were collected every minute during the course of the run. Subsequently, all the 24 collected fractions were mixed, lyophilized and kept at −80°C prior to Nano-LC- MS/MS analyses.

### Nano-LC- MS/MS analyses

The collected fractions were examined by nano-RPLC-MS/MS using a Dionex's Ultimate 3000 RSLCnano system (Dionex, Sunnyvale, CA) coupled to a Thermo Scientific Q Exactive mass spectrometer equipped with a NanoFlex source (Thermo Fisher Scientific). A capillary RP-LC column (75 um i.d. x 150mm, filled with Acclaim PepMap RSLC C18, 100Å, 2 um, nanoViper Dionex, Sunnyvale, CA) was used for separation of peptides by LC. First, samples were desalted from the autosampler at 5 μL/min by loading onto a trap column (Acclaim PepMap 100 C18, 100 Å, 3 μm, 75 μm×2 cm, Dionex, Sunnyvale, CA). Thereafter, desalted samples were washed for 12 min with 0.1% FA in HPLC grade water and the system changed into line with the analytical RP capillary column. The tryptic digest was analyzed within 65 min 3-step gradient (80% ACN in 0.1% methanol from 4% to 50% over 45 min, 50% to 90% over 5 min and kept at 95% for 15 min) at a flow rate of 300 nL/min. Key parameter settings for the Thermo Scientific Q Exactive mass spectrometer were as follows:

- First grade MS parameters: Resolution: 70,000, AGC target: 3e6, Maximum IT: 0 ms, Scan range: 350 to 1800 m/z.
- Second grade MS parameters: Resolution: 17,500, AGC target: 1e5, Maximum IT: 60 ms, TopN: 20, NCE / stepped NCE: 30

### Database search for protein identification

The protein identification was carried out using the Paragon^TM^ algorithm as the search engine in the ProteinPilot^TM^ software (version 5.0; Applied Biosystems). Each of the RAW files created from the MS system was converted to a ProteinPilot compatible Mascot Generic Format (MGF) with preselected iTRAQ reporter ions. The MGF files were searched against the *Homo sapiens* database in UniProt along with contaminant protein sequences in December 2015 using ProteinPilot™ Software 5.0 (AB Sciex) and the following search parameters: Sample Type: iTRAQ 6-plex (Peptide Labeled); Cys-alkylation: MMTS; Digestion: Trypsin; ID focus: Biological modification; Use FDR analysis; Thorough search and an Unused Protscore (Conf)): ≥1.3%.

### Calculation of iTRAQ differential ratios

For computing the differential ratio, the data of each iTRAQ-labeled sample from the IPF or control group was compared with the data from the other five iTRAQ labeled samples. Summary with iTRAQ ratios (with control samples as the denominator for determining fold change) was evaluated using a workflow built within Galaxy-P [27] to yield UniProt accession numbers and gene names of identified proteins. During the quantification process, we included exclusively peptides that were unique for a given protein and proteins with a minimum number of at least one peptide having an ion score greater than 99% confidence. On the contrary, identified peptides with low confidence score of 1%, those without peaks corresponding to iTRAQ labels and those found in multiple proteins were excluded. The quantitative estimates computed by ProteinPilot for each protein included: differential expression fold change ratios; the *p* value and the error factor (EF = 10^95% CI^ where 95% CI = (ratio × EF) − (ratio/EF)). The false discovery rate (FDR) was computed using the decoy database search approach by alignment of the spectra against the *Homo sapiens* decoy database. After identification and quantification, the data were exported into an Excel file format for further screening analysis.

### Analysis of differentially expressed proteins

After identification and quantification steps using ProteinPilot, proteins identified at 1% FDR with a confidence threshold of 95% (Proteinpilot Unused score ≥1.3) in all runs and *p*-values < 0.05 in at least one pairwise comparison were retained. The volcano plot of fold-change ratios as a function of *p*-values was generated using GraphPad Prism 6.01 for windows. The data discrepancy in iTRAQ experiments usually originates from the following three aspects: technical, experimental, and biological variations. In biological and technical repeatability analyses, the most commonly used statistical model is the Coefficient of variation (CV) which refers to the degree of dispersion between the data sizes. Therefore, we used the CV to assess the reproducibility of biological or technical replicates. The smaller the CV, the smaller the risk and, on the contrary, the greater the CV, the greater the risk. Usually data with CV ≤ 50% can be considered to be within the acceptable range. For screening the differentially expressed proteins, two statistical approaches were used. In the first approach proteins with the CV ≤ 0.5 were discriminated according to the average ratio AVG. Proteins with AVG ≥ 1.5 or ≤ 0.67 were respectively considered as up-regulated and down-regulated proteins. The second approach no longer considered the CV and AVG values but directly screened out proteins with fold change ratio ≥ 1.5 or ≤ 0.67 in each pair group as downregulated and upregulated proteins, respectively. This approach is more stringent and can ensure that the results of each group are in line with the requirements, but may filter out some meaningful proteins.

### Construction of IFP regulatory network and functional enrichment analyses

The set of differentially expressed proteins was mapped by the online Search Tool for the Retrieval of Interacting Genes (STRING) database (http://string-db.org) to disclose possible connections among proteins and visualize the PPI (protein-protein interaction) network. The PPI network was constructed by setting the minimum required interaction score to medium confidence (0.4). The active interaction sources included were “experiments” and “textmining”. The setting parameters for maximum number of interactors to show for first shell and second shell were respectively “none/query protein only” and “none”. Gene Ontology (GO) functional enrichment analyses of proteins in the PPI network was directly performed online to retrieve GO terms assigned to a set of proteins in the GO categories of molecular function, biological process and cell component with a false discovery rate (FDR) cutoff of < 0.05 on the whole genome background.

### ELISA test for validation of biomarkers

To validate the iTRAQ data, four most up-regulated and three most down-regulated proteins were selected as candidate proteins for the validation phase by ELISA test based on their expression levels. Protein concentrations in the serum samples were assayed using human hemoglobin beta (ab157707), C reactive protein (CRP) (ab99995), alpha 1 antitrypsin (SERPINA1) (ab108799), apolipoprotein AII (APOA2) (ab108805), fetuin A (AHSG) (ab108855), kininogen (KNG) (ab108876) and alpha 1 microglobulin (ab108884) ELISA kits purchased from ABCAM (Abcam, Cambridge, UK) according to the manufacturer’s protocols provided with each kit.

### Statistical analysis

One way ANOVA with Bonferoni multiple comparison post-test was used to evaluate the statistical significance during the validation step. Receiver Operating Characteristic (ROC) curves were generated for each selected differentially expressed protein in the validation experiment using GraphPad Prism software. If the area under the ROC (AUC) was superior to 0.7, the selected protein was considered as a significantly informative IPF biomarker.

## Results

### Proteins identified by peptide spectral matching and database searching

**Fig 1:**
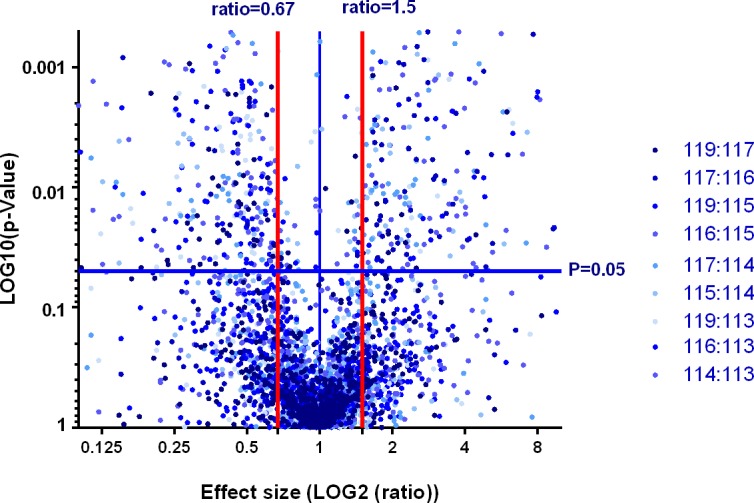
Volcano plot showing log2 fold change plotted against log10 adjusted P value for IPF samples versus healthy control samples. Data points in the upper right (ratio > 1.5) and upper left (ratio < 0.67) sections with P>0.05 represent proteins that are significantly dysregulated in IPF patients according to the Protein Pilot analysis of the six-plex iTRAQ-labelled serum samples (3 groups of healthy individuals labelled 113, 115 and 117 and 3 groups of IPF patients labelled 114, 116 and 119). The Volcano plot was generated using GraphPad Prism software version 6.01 for windows.

The Protein Pilot was used to generate the summary output files of the 2D LC-MS analyses of the six-plex iTRAQ-labelled serum samples (3 groups of healthy individuals labelled 113, 115 and 117 and 3 groups of IPF patients labelled 114, 116 and 119) and proteins were identified at 1% FDR in accordance with AB SCIEX official document. The "iTRAQ Search Results" are reported in S1–S6 Files, each of which has two sub-tables. The first sub-table contains the credible protein screening result while the second sub-table stands for the original search results. Each file represents the relative protein ratios obtained by comparison of one of the iTRAQ tag with other tags. For example, S1 File represents the relative ratios obtained by comparison of all the groups (114, 115, 116, 177 and 119) with the proteins of the 113 label group. The Volcano plot of the p-values generated by Protein pilot in function of the protein ratio obtained by comparison of healthy and IPF patients is depicted in Fig 1. After completion of protein search, proteins with unused ≥ 1.3 at confidence interval CI > 95% and p-values < 0.05 were retained while misidentified proteins, suspected contaminants and the reverse matches that occurred in the reverse library (peptide with "RRRRR" in the beginning of the decoy) were manually removed. After removal of these proteins, a total of 490 credible proteins was obtained. Thereafter, the coefficient of variation (C.V) was used to evaluate the reproducibility of the pairwise comparison between labeled samples. The results of the reproducibility analysis of the iTRAQ labeling data showed that 394 proteins (more than 80.4% of the credible proteins) could be recovered at C.V ≤ 50%.

### Identification of differentially expressed proteins

To identify differentially expressed proteins, two methods were employed. The screening results are reported in S7 File. In the first approach, only the 394 proteins confident proteins with C.V ≤ 50% were used as background for screening upregulated (AVG≥1.5) and down-regulated (AVG≤0.67) proteins. This method allowed the identification of 32 upregulated (C.V≤0.5, AVG≥1.5) and 58 downregulated (C.V≤0.5, AVG≤0.67) proteins. In the second screening method, the set of 490 credible proteins was used as background to screen proteins with ratio ≥1.5 as upregulated proteins (10 proteins) and those with ratio ≤ 0.67 as downregulated proteins (22 proteins) in pairwise comparisons without considering C.V and AVG. Merging the results of both methods showed that a total of 97 proteins, including 38 upregulated (AVG≥1.5) and 59 downregulated (AVG≤0.67) proteins, displayed consistent differential expression when comparing serum samples from IPF patients and healthy controls. Four upregulated and 21 downregulated proteins were common differentially expressed proteins identified when using both methods. The list of screened differentially expressed proteins is shown in S1 File (excel sheet “Total DEP). The six most upregulated differentially expressed proteins among the 38 identified were hemoglobin subunit beta (HBB), C-reactive protein (CRP), alpha-1-antitrypsin (SERPINA1), pulmonary surfactant-associated protein B (SFTPB), haptoglobin (HP) and fibrinogen beta chain (FGB) while the most downregulated differentially expressed proteins included apolipoprotein A-II (APOA2), alpha-2-HS-glycoprotein (AHSG), kininogen-1 (KNG1) and protein AMBP (AMBP).

### Bioinformatics analyses of differentially expressed proteins

**Fig 2:**
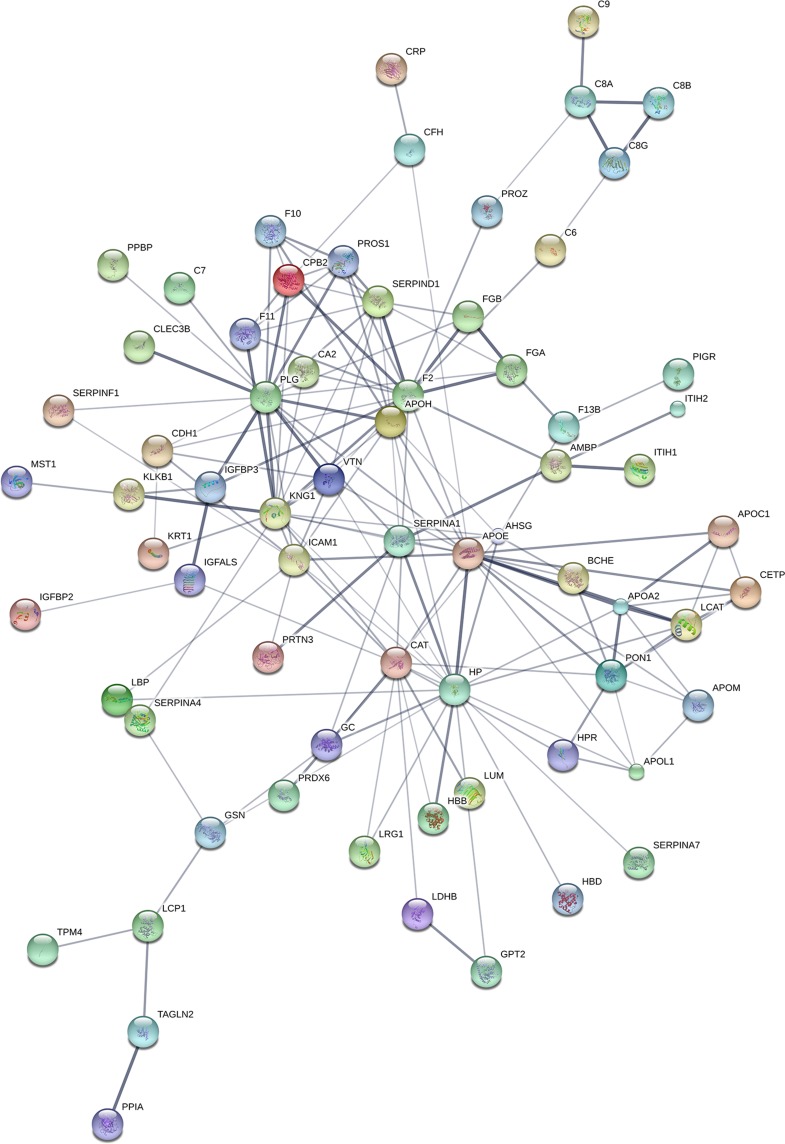
Protein-protein interaction regulatory network of proteins differentially expressed between IPF and healthy controls. Differentially expressed proteins were combined for building a regulatory network using String software. The network contained 87 nodes and 243 edges with an average node degree of 5.01 and a clustering coefficient of 0.655. The PPI enrichment *p*-value was equal to 0.

To further our understanding on the regulatory mechanisms of screened differentially expressed proteins and find out their potential role in IPF, we combined down-regulated and up-regulated proteins for building a regulatory network using String software. The result (Fig 2) demonstrated that the screened differentially expressed proteins form a complex regulatory network containing 87 nodes and 243 edges with an average node degree of 5.01 and a clustering coefficient of 0.655. The number of expected edges was 12, which was less than the actual 243 edges found, and the PPI enrichment p-value was 0. This result indicates that the network had significantly more interactions than expected, which means that the proteins are at least partially biologically connected as a group. PLG, KNG1 and F2 were found as the most important hubs implicated in gene regulation in the constructed IPF network.

**Fig 3:**
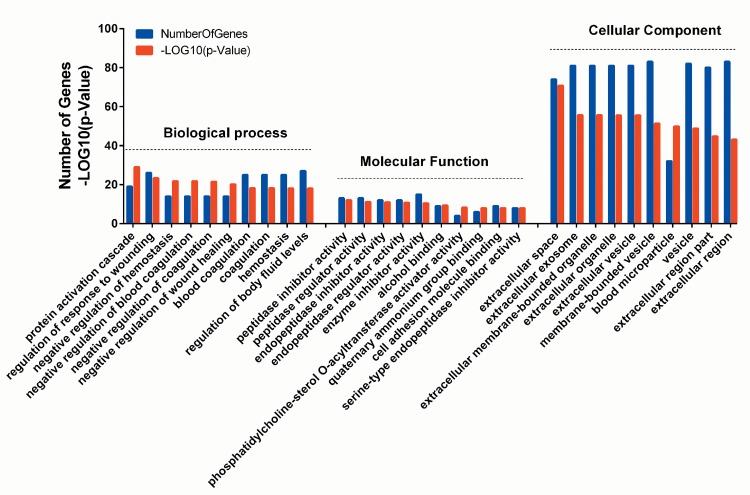
GO enrichment of differentially expressed proteins. The functional enrichment of proteins in the constructed interaction network was carried out online in the STRING database. Only the 10 most significantly enriched GO terms in each GO category (Biological Process, Cellular Component and Molecular function) with their p-values were presented. A GO term was considered significant at p-value < 0.05.

Functional enrichment in the network revealed that the 97 differentially expressed proteins were involved in 287 significant functional terms (p-value < 0.05) in the category of “biological process”, 44 in the category of “molecular function” and 46 in the category of “cellular component”. The functional terms in each category, ranked by statistical significance, were summarized in S2 File. As presented in Fig 3, the top significantly enriched GO terms in the category of “biological process” included protein activation cascade (17 proteins), regulation of response to wounding (24 proteins), negative regulation of blood coagulation (13 proteins), negative regulation of coagulation (13 proteins) and negative regulation of wound healing (13 proteins) with 44 of these proteins participating in response to stress pathway. For “molecular function”, peptidase inhibitor activity (12 proteins), peptidase regulator activity (12 proteins) and endopeptidase inhibitor activity (11 proteins) were the most significant terms. Protein binding and hydrolase activity were respectively enriched with 36 and 20 proteins. In the category of “cellular components”, extracellular space (68 proteins), extracellular organelle (73 proteins) and extracellular membrane-bounded organelle (73 proteins) were the most significantly enriched terms. More than 75 of the proteins were implicated in extracellular components.

### Validation of identified biomarkers by ELISA

**Fig 4:**
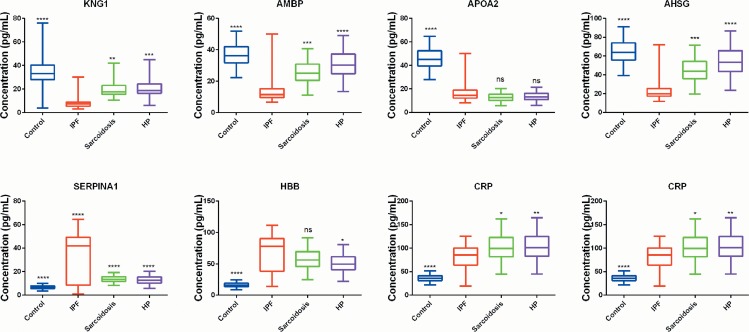
Box plots of levels of proteins selected for the validation experiment. Levels of these selected biomarkers were determined in serum of healthy controls (n = 20), sarcoidosis (n = 20), hypersensitivity pneumonitis (HP) (n = 20), and IPF patients (n = 20) using ELISA, n, number of subjects. P-values were calculated with one-way ANOVA test. *p<0.05, **p<0.01, ***p<0.001 and ****p<0.0001 compared to IPF group.

Validation of candidate protein biomarkers is vital for moving from the initial discovery to possible applications. A set of three most significantly upregulated proteins (HBB, CRP and SERPINA1) and four most significantly down-regulated proteins (APOA2, AHSG, KNG1 and AMBP) were selected for verification in validation cohorts (n = 80, 20 healthy controls, 20 IPF patients, 20 hypersensitivity pneumonitis patients and 20 sarcoidosis patients) using ELISA. As displayed in Fig 4, the serum concentrations of HBB, CRP and SERPINA1 in IPF, sarcoidosis and hypersensitivity pneumonitis patients were higher compared to the healthy control group. On the contrary the levels of APOA2, AHSG, KNG1 and AMBP were lower in the IPF group. There were significant differences between the concentrations of HBB, AHSG, KNG1, SERPINA1 and AMBP in the IPF group and those in the other groups. No statistical difference was recorded on the concentrations of CRP and APOA2 among the IPF, sarcoidosis and hypersensitivity pneumonitis groups. In addition, significant differences were observed between healthy control and sarcoidosis and hypersensitivity pneumonitis groups concerning the levels of the set of proteins selected for validation purposes. In addition, the ELISA validation results confirmed the iTRAQ-LC-MS/MS findings. These results suggested that HBB, AHSG, KNG1, SERPINA1 and AMBP could be specific biomarkers for diagnosis of IPF.

### ROC analysis

**Fig 5:**
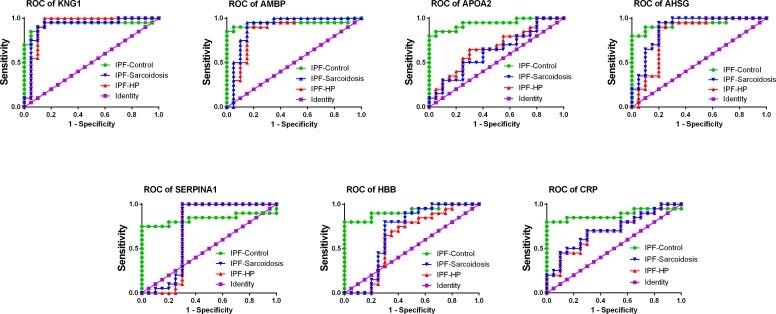
ROC curve analysis of validated differentially expressed proteins. Accuracy of selected candidate biomarkers in discriminating IPF from healthy controls, sarcoidosis and HP was evaluated using receiver operating characteristic (ROC) curves. The area under the ROC curve AUC > 0.7 was considered in deciding if a given biomarker was informative in discriminating compared groups from each other.

ROC analysis (Fig 5) was performed to assess the biomarker potential of validated differentially expressed proteins. The diagnostic accuracy of each biomarker among disease groups is reported in Table 2. We found that in the testing of AMBP as a biomarker, the areas under the ROC curve (AUC) were 0.9425, 0.8650 and 0.9025, respectively, to discriminate between IPF and healthy controls, IPF and sarcoidosis, and IPF and hypersensitivity pneumonitis. When the individual protein AHSG served as a biomarker, the AUCs were 0.9400, 08250 and 0.8900, respectively, to discriminate between IPF and healthy controls, IPF and sarcoidosis, and IPF and hypersensitivity pneumonitis. Similarly, assessment of SERPINA1 led to diagnostic accuracy (expressed in AUC) of 0.8375, 0.7050 and 0.7200, respectively, to discriminate between IPF and healthy controls, IPF and sarcoidosis, and IPF and hypersensitivity pneumonitis. In addition, CRP showed AUCs of 0.8825, 0.7000 and 0.7100 for discriminating IPF and healthy controls, IPF and sarcoidosis, and IPF and hypersensitivity pneumonitis, respectively. HBB and APOA2 could help discriminate between IPF and healthy controls, but was unable to be applied for differential diagnostic of IPF and sarcoidosis, and IPF and hypersensitivity pneumonitis, respectively. Besides, KNG1 could serve as a biomarker for discriminating between IPF and healthy controls and IPF and hypersensitivity pneumonitis, but could not help discriminate between IPF and sarcoidosis. Overall, the results showed that AHSG, CRP, SERPINA1, AMBP and in some degree, KNG1, exhibited potentiality to serve as IPF biomarkers. The ELISA data supported the iTRAQ results in some extent.

**Table 2:** Results of ROC curve analysis of validated differentially expressed proteins. AUC: Area Under the ROC Curve.

		KNG1	AMBP	APOA2	AHSG	SERPINA1	HBB	CRP
**IPF-Control**	**AUC**	0.9325	0.9425	0.9425	0.94	0.8375	0.92	0.8825
	**Std. Error**	0.04847	0.04532	0.03743	0.03945	0.07434	0.04549	0.06245
	**95% confidence interval**	0.8375 to 1.028	0.8537 to 1.031	0.8691 to 1.016	0.8627 to 1.017	0.6918 to 0.9832	0.8308 to 1.009	0.7601 to 1.005
	**P-value**	< 0.0001	< 0.0001	< 0.0001	< 0.0001	0.0003	< 0.0001	< 0.0001
**IPF-Sarcoidosis**	**AUC**	0.9275	0.865	0.6675	0.825	0.705	0.6275	0.7
	**Std. Error**	0.04923	0.06445	0.08596	0.07362	0.101	0.09488	0.08315
	**95% confidence interval**	0.8310 to 1.024	0.7387 to 0.9913	0.4990 to 0.8360	0.6807 to 0.9693	0.5070 to 0.9030	0.4415 to 0.8135	0.5370 to 0.8630
	**P-value**	< 0.0001	< 0.0001	0.0699	0.0004	0.0265	0.1677	0.0305
**IPF-HP**	**AUC**	0.91	0.9025	0.6225	0.89	0.72	0.685	0.71
	**Std. Error**	0.05443	0.05604	0.08909	0.05512	0.09681	0.09353	0.08217
	**95% confidence interval**	0.8033 to 1.017	0.7926 to 1.012	0.4478 to 0.7972	0.7819 to 0.9981	0.5302 to 0.9098	0.5016 to 0.8684	0.5489 to 0.8711
	**P-value**	< 0.0001	< 0.0001	0.185	< 0.0001	0.0173	0.0453	0.0231

## Discussion

Molecular biomarkers are highly desired in idiopathic pulmonary fibrosis (IPF) where they hold the potential to elucidate underlying disease mechanisms, accelerated drug development, and advance clinical management [8]. At present, there are no molecular biomarkers made available in well-known clinical application for IPF, and the research for discovery of potential markers remains in its early stages. Therefore, screening differentially expressed serum proteins in patients with IPF could provide an effective way to identify diagnostic biomarkers. In previous studies, a set of IPF biomarkers have been developed by applying proteomics, transcriptomics and genomics approaches to the analysis of lung tissue, bronchoalveolar lavage fluid (BALF) or serum for patients with IPF. For instance, high levels of matrix metalloproteinase-8 (MMP-8), pepsin, BALF YKL-40, S100A9 and MRP14 have been found elevated in the BALFs of IPF patients [15, 28–31]. Similarly, elevated serum levels of vascular endothelial growth factor (VEGF), MMP3, CXCL13, heat shock protein 47 (HSP47) and several other proteins were equally reported in IPF [7, 13, 15, 30, 32–42]. In the present study, iTRAQ labeling coupled with 2D LC-MS/MS analysis was used to identify differentially expressed proteins in serum of IPF patients. A total of 97 differentially expressed proteins were identified and eight different proteins including HBB, CRP, SERPINA1, APOA2, AHSG, KNG1 and AMBP were validated by ELISA. Finally, we evaluated the diagnostic accuracy of validated differentially expressed proteins and found that AHSG, CRP, SERPINA1, AMBP and KNG1 may be potential specific diagnostic biomarkers for IPF. In the present work, CRP was found as the most upregulated protein in the serum of IPF patients but its application as IPF biomarker still constitutes a problematic since it has been associated to various diseases such as chronic obstructive lung disease and lung cancer, obliterative bronchiolitis and systemic lupus erythematosus [43–47]. The functional role of APOA2, the most downregulated differentially expressed proteins identified in the present study, has not been reported in IPF in the past. However, low concentrations APOA1 were found in bronchoalveolar lavage fluids from subjects with IPF [48]. This suggested the involvement of APOA2 in the pathophysiology of IPF. In this study, we found hemoglobin (HBB) as a biomarker for IPF. This should be taken with precaution because hemoglobin is generally regarded as a marker for lysis of erythrocytes during sample draw [49]. Intriguingly, most of previously reported IPF biomarkers [7–16, 28–42, 50–70] were not identified in our study. Therefore, the present findings are promising for the discovery and development of novel biomarkers for IPF.

In order to verify the interactions between the differentially expressed proteins, a regulatory network was constructed. The results showed that PLG, KNG1 and F2 were the most important hubs orchestrating protein regulation in the constructed IPF regulatory network. The downregulation of PLG in IPF was in corroboration with previous findings demonstrating the anti-fibrotic and anti-apoptotic roles of plasminogen activator inhibitor-1 in fibroblasts from fibrotic lungs [71, 72]. The expressions of KNG1 and F2 in IPF have not been investigated before. Further studies are needed to validate these proteins as well as the remainders of differentially expressed proteins as serum biomarkers for IPF.

The GO functional analysis revealed that the differentially expressed proteins were majorly implicated in the extracellular components, coagulation and in response to wounding, each of which is a fundamental element in IPF.

## Conclusions

In conclusion, the use of iTRAQ labeling of serum samples from IPF patients in combination with LC-MS/MS allowed the discovery of numerous novel, differentially expressed proteins between IPF patients and normal controls. A preliminary analysis of the specificity of some of these proteins exhibited good discriminatory power against other lung diseases. The new potential biomarkers warrant further in-depth experimentation to be validated as IPF biomarkers.

## Supporting Information

S1 File Relative ratios obtained by comparison of all the groups (114, 115, 116, 177 and 119) with data stemmed from the 113 label. (XLSX)

S2 File Relative ratios obtained by comparison of all the groups (113, 115, 116, 177 and 119) with data stemmed from the 114 label. (XLSX)

S3 File Relative ratios obtained by comparison of all the groups (114, 113, 116, 177 and 119) with data stemmed from the 115 label. (XLSX)

S4 File Relative ratios obtained by comparison of all the groups (113, 114, 115, 177 and 119) with data stemmed from the 116 label. (XLSX)

S5 File Relative ratios obtained by comparison of all the groups (113, 114, 115, 116 and 119) with data stemmed from the 177 label. (XLSX)

S6 File Relative ratios obtained by comparison of all the groups (113, 114, 115, 116, 177 and) with data stemmed from the 119 label. (XLSX)

S7 File Screening of differentially expressed proteins from the iTRAQ-LC/MS/MS data. (XLSX)
